# Prioritizing guideline recommendations for implementation: a systematic, consumer-inclusive process with a case study using the Australian Clinical Guidelines for Stroke Management

**DOI:** 10.1186/s12961-021-00734-w

**Published:** 2021-05-22

**Authors:** Elizabeth A. Lynch, Chris Lassig, Tari Turner, Leonid Churilov, Kelvin Hill, Kirstine Shrubsole

**Affiliations:** 1 Caring Futures Institute, College of Nursing and Health Sciences, Flinders University, Sturt Campus, GPO Box 2100, Adelaide, SA 5001 Australia; 2 Adelaide Nursing School, University of Adelaide, Level 4 AHMS Building, Adelaide, 5005 Australia; 3 NHMRC Centre of Research Excellence in Stroke Rehabilitation and Brain Recovery, 245 Burgundy St, Heidelberg, VIC 3084 Australia; 4 Stroke Foundation, Level 7/461 Bourke St, Melbourne, VIC 3000 Australia; 5 Cochrane Australia, Level 4/553 St Kilda Rd, Melbourne, VIC 3004 Australia; 6 Melbourne Medical School, University of Melbourne, Parkville, VIC 3010 Australia; 7 Southern Cross University, Bilinga, QLD 4225 Australia; 8 The Queensland Aphasia Research Centre, The University of Queensland, Brisbane, QLD Australia; 9 Centre of Research Excellence in Aphasia Recovery and Rehabilitation, Bundoora, Australia

**Keywords:** Guidelines as topic, Implementation science, Patient preference, Health services, Stroke

## Abstract

**Background:**

Implementation of evidence-based care remains a key challenge in clinical practice. Determining “what” to implement can guide implementation efforts. This paper describes a process developed to identify priority recommendations from clinical guidelines for implementation, incorporating the perspectives of both consumers and health professionals. A case study is presented where the process was used to prioritize recommendations for implementation from the Australian Stroke Clinical Guidelines.

**Methods:**

The process was developed by a multidisciplinary group of researchers following consultation with experts in the field of implementation and stroke care in Australia. Use of the process incorporated surveys and facilitated workshops. Survey data were analysed descriptively; responses to ranking exercises were analysed via a graph theory-based voting system.

**Results:**

The four-step process to identify high-priority recommendations for implementation comprised the following: (1) identifying key implementation criteria, which included (a) reliability of the evidence underpinning the recommendation, (b) capacity to measure change in practice, (c) a recommendation–practice gap, (d) clinical importance and (e) feasibility of making the recommended changes; (2) shortlisting recommendations; (3) ranking shortlisted recommendations and (4) reaching consensus on top priorities.

The process was applied to the Australian Stroke Clinical Guidelines between February 2019 and February 2020. Seventy-five health professionals and 16 consumers participated. Use of the process was feasible. Three recommendations were identified as priorities for implementation from over 400 recommendations.

**Conclusion:**

It is possible to implement a robust process which involves consumers, clinicians and researchers to systematically prioritize guideline recommendations for implementation. The process is generalizable and could be applied in clinical areas other than stroke and in different geographical regions to identify implementation priorities. The identification of three clear priority recommendations for implementation from the Australian Stroke Clinical Guidelines will directly inform the development and delivery of national implementation strategies.

**Supplementary Information:**

The online version contains supplementary material available at 10.1186/s12961-021-00734-w.

## Background

Despite the widespread availability of clinical practice guidelines for different health conditions, it is widely acknowledged that translation of evidence-based guideline recommendations into improved clinical practice is challenging, with complex barriers at multiple levels. While there is some evidence that dissemination of guidelines can promote the use of evidence-based practice, guideline impact is enhanced substantially when accompanied by tailored, well-resourced implementation strategies [1].

A key step in implementing evidence-based guideline recommendations is to determine what exactly should be implemented, or as Lavis et al. have described, what should be transferred [2]. In a healthcare environment where there are multiple competing demands for finite resources, potential service improvements need to be prioritized from a large set of evidence-based recommendations. Methods for identifying priorities for implementation are in the early stages of development [3], and there is no consensus on the best approach for prioritizing implementation activities. It is important to know the preferences of both consumers and health professionals, because patient and family expectations can influence change [4, 5], and local opinion leaders and existing culture can influence implementation outcomes [6, 7]. But again, there is little practical guidance on how to incorporate and synthesize the implementation priorities of both consumers and health professionals in a rigorous and feasible way.

In order to be accountable to funding bodies, government agencies and other stakeholder groups affected by, or responsible for, implementation efforts, it is important to have transparent, rigorous and reproducible processes when setting national implementation priorities. The priority-setting process should be able to incorporate the perspectives of different stakeholder groups, account for different dimensions or values important to these stakeholder groups, and have capacity to be applied to a large number of recommendations.

## Methods

This purpose of this paper is to present a step-by-step process developed specifically to systematically prioritize guideline recommendations for implementation, which incorporates input from consumers and health professionals. The process was developed by a multidisciplinary team comprising health professional researchers, guideline developers, statisticians and implementation scientists. We developed this process to allow us to identify priorities for implementation in the field of stroke in Australia, as we were unable to identify methods that suited our aims in the published literature. We provide a case study to illustrate how the process was used to select the top three priority recommendations for implementation from the Australian Stroke Guidelines. These priority recommendations will be addressed through the development and delivery of national implementation strategies in a subsequent piece of work.

## Case study: the Australian stroke context

In 2020, 27,400 Australians experienced a first-ever stroke, and there were an estimated 445,000 people living with stroke in Australia [8]. Australia has well-developed stroke healthcare systems, with nationally endorsed “living” stroke clinical practice guidelines (hereafter referred to as Stroke Guidelines) in which evidence surveillance occurs monthly, relevant literature is reviewed to determine potential impact to current recommendations, and recommendations are reviewed when assessments indicate a potential impact [9]. In addition, there is a well-established national stroke audit programme [10] and a stroke clinical registry with over 100,000 registrants [11]. The Stroke Guidelines contain over 400 evidence-based clinical practice recommendations grouped into eight chapters (for example pre-hospital care, acute medical and surgical management, secondary prevention, rehabilitation) and are freely available online (https://informme.org.au/en/Guidelines/Clinical-Guidelines-for-Stroke-Management).

Details on the process used to develop and maintain the living format of the Stroke Guidelines is available in Additional file 1: Appendix 1.

**Fig. 1:**
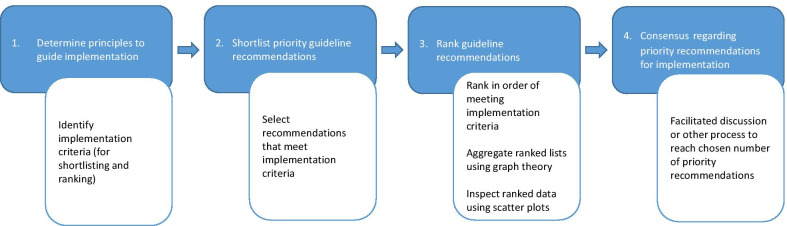
Guideline prioritization process

The implementation prioritization process consisted of the establishment of key implementation criteria, followed by a series of activities to shortlist guideline recommendations, and finally a consensus process (see Fig. 1).

### Step 1: identifying key implementation criteria

Factors associated with the implementation of evidence-based care can vary between geographical regions and different health conditions. Therefore, experts familiar with the local setting, health evidence and knowledge about evidence implementation should identify the principles that will guide each guideline prioritization project.

## Case study: expert panel identifying key implementation criteria

A Stroke Guidelines Knowledge Translation Expert group was convened by Cochrane Australia and the Stroke Foundation in late 2018. This group comprised an expert panel of 14 researchers with expertise in implementing stroke guideline recommendations (Including authors EAL and KS), two project team members from Cochrane Australia (including author TT) and two project team members from the Stroke Foundation (authors CL and KH). Thirteen of the 14 expert panel members had worked as health professionals in the field of stroke (disciplines included nursing, neurology, rehabilitation medicine, physiotherapy, occupational therapy and speech pathology) and had 15 or more years of experience in the stroke field; all had led stroke-specific research projects. Ten expert panel members were involved in guideline development, being named on the Living Guidelines Executive Project Group, the content steering committee, or a content working group (detail of Living Guidelines working groups can be found at https://informme.org.au/Guidelines/Clinical-Guidelines-for-Stroke-Management/Guidelines-supporting-documents).

The project team members facilitated a full-day workshop in February 2019, attended by 12 of the 14 expert panel members. The expert panel was tasked with determining the principles to guide prioritization of recommendations for implementation from the Stroke Guidelines. In this workshop, the expert panel reached a consensus that recommendations should be prioritized according to the following criteria:

1. Reliability of the evidence underpinning the recommendation, leading to a degree of certainty in the recommendation being made
2. Extent to which it is possible to determine current levels of practice, and to measure any changes in the practice resulting from implementation activities
3. The existence and size of the gap between recommended and actual practice, as identified in national stroke data collection programmes
4. Clinical importance, as judged by both consumers and clinicians
5. Feasibility of making the recommended changes, and the existence of associated impacts on other elements of the health practice and policy environment

### Step 2: shortlisting priority guideline recommendations

Criteria that are clearly defined can be applied by the project team to shortlist recommendations prior to consulting with different stakeholder groups. This then eliminates recommendations that are lower priorities (in terms of not meeting the recommended criteria) from further consideration.

Criteria to guide prioritization that are inherently subjective in nature (for example “important”, “easy”, or “feasible”) can be shortlisted by stakeholders who will be affected by, or responsible for, implementation of priority recommendations. We would recommend that at a minimum, health professionals and people living with the health condition should be involved. Data from the different stakeholder groups should be analysed separately.

Stakeholders can rate how well each recommendation aligns with the implementation criteria (e.g. on a scale of 1–10, how *[insert criteria*] is this recommendation?). Descriptive statistics (medians, means) can be used to shortlist the recommendations to a number deemed workable to proceed to the ranking exercise.

## Case study: project team shortlisting priority guideline recommendations according to *reliability of evidence* and *capacity to measure change in practice*

Each Stroke Guideline recommendation is accompanied by a strength rating (strong or weak), as assessed using the GRADE (Grading of Recommendations, Assessment, Development and Evaluation) methodology [12] (See Additional file 1: Appendix 1 for more details on how GRADE was used in Stroke Guideline development). Strong Stroke Guideline recommendations are those that Guideline developers are confident are supported by evidence of a clear balance towards either desirable or undesirable effects. Therefore, an overall recommendation rating of “strong” was used as a proxy for recommendations underpinned by reliable evidence. Author CL reviewed the published strength rating of all recommendations in the Stroke Guidelines and identified 92 strong recommendations.

Capacity to measure change in practice for each of the 92 strong recommendations was assessed by authors CL and KH by reviewing whether data were routinely collected on these recommendations in either the acute or rehabilitation national stroke audits, or via the Australian Stroke Clinical Registry. Thirty-two recommendations met both criteria of being underpinned by reliable evidence and having the capacity for practice to be measured with current routine data collection methods.

Authors EL, CL and KH reviewed these 32 strong, measurable recommendations, and bundled recommendations that are delivered as packages of care. This resulted in a shortlist of 23 (single or bundled) recommendations.

## Case study: wider stroke community shortlisting priority guideline recommendations according to *importance*

The shortlist of 23 recommendations was further refined by conducting an online survey using SurveyMonkey (see Additional file 2: Appendix 2), administered from January to February 2020. Eligible participants were stroke survivors and their family members or carers who were members of the consumer panel of the Stroke Foundation Living Guidelines project, and clinicians or researchers working in the field of stroke, recruited via emailed invitations to participate sent via the Australian Stroke Coalition and state-based stroke clinical networks. In the survey, each recommendation was presented with a lay description, the proportion of people impacted by the recommendation and the potential impact of implementing the recommendation (details of proportion and impact as reported in the National Stroke Audit and the Stroke Guidelines website, respectively). Participants were invited to rate the relative importance of each of the 23 recommendations on a scale from 1 to 10 (not important to extremely important), and to select their top five implementation priorities. Participants could also nominate recommendations that were not included in the survey but that they considered were priorities for implementation.

Data from the survey were exported to an Excel spreadsheet, and responses from clinicians/researchers and stroke survivors/carers/family members were analysed separately. The 10 most important recommendations were identified by reviewing the median importance scores for each recommendation, and the frequency in which the recommendation was ranked as a top five recommendation from the consumer and health professional groups. Data were sorted to rank recommendations in order of importance (median, top 5) for each group.

### Step 3: ranking guideline recommendations

The penultimate step in the prioritization process entails individuals from the relevant stakeholder groups ranking the shortlisted recommendations relative to other recommendations, that is, “rank recommendations in order of most [*implementation criteria*] to least [*implementation criteria*]”. When there are two implementation criteria to consider, the relative ranking of recommendations can be compared by aggregating each individual’s ranked responses into a ranked list for the whole group using a graph theory-based voting system implemented as a decision support tool in Microsoft Excel [13]. A scatter plot can facilitate visual interpretation of the relative ranking of the recommendations in terms of the two implementation criteria. This process can be done numerous times if there are more than two implementation criteria to consider. Individual data points on the scatter plot represent the different recommendations, with one criterion (criterion A) on the horizontal axis and the second criterion (criterion B) on the vertical axis (higher values represented higher rankings in terms of closer alignment with the implementation criteria). Therefore, a hypothetical recommendation that is ranked highest on both criterion A and criterion B would be located at the right top corner of the plot. For a given recommendation, all recommendations located higher on the plot would be ranked higher on criterion A and all recommendations located to the right on the plot would be more highly ranked on criterion B.

Recommendations that are ranked lower than other recommendations on both criteria are lower priorities for implementation.

## Case study: wider stroke community ranking guideline recommendations according to *importance* and *feasibility*

A second survey was emailed in mid-February to participants from survey 1 who had provided their contact details. The top 10 recommendations from survey 1 were presented alongside information about the proportion of people affected and likely impact of each recommendation. Participants were asked to rank recommendations in order of most important to least important. Health professionals were also asked to complete a second ranking activity to order recommendations from most feasible to least feasible to implement.

Author LC aggregated the responses into a ranked list for the whole group using a graph theory-based voting system in Microsoft Excel. Author EL entered these data into a scatter plot with importance on the horizontal axis and feasibility on the vertical axis (higher values represented higher importance and feasibility.

### Step 4: reaching consensus on priority recommendations for implementation

The final stage is to reach consensus on the priority recommendations for implementation. Representatives from key stakeholder groups (i.e. those who will be affected by, or responsible for, implementation of priority recommendations) should be involved. The format of the consensus-seeking forum should allow for open and respectful discussion, with a clear aim of the forum to reach consensus on the set number of priority recommendations.

## Case study: reaching consensus

The consensus phase involved a facilitated workshop with stroke consumers and health professionals to decide upon the top three priority recommendations for implementation. The workshop was held via Zoom videoconference rather than the originally planned face-to-face meeting due to the COVID-19 pandemic. Participants who completed the surveys and volunteered to be involved in the consensus meeting were then purposively invited to participate in the facilitated workshop so that there was representation from stroke survivors, carers, researchers and health professionals from different disciplines and different regions.

Prior to the workshop, attendees were asked to prepare by reading a document and viewing a 5-minute video which provided an overview of the project and work to date. In the workshop, results from surveys 1 and 2 were summarized, and the top 10 priority recommendations presented. Information about the current evidence–practice gap for each of the top 10 recommendations was provided, derived from the Stroke Foundation national audit and national benchmarking targets. Attendees were then invited to discuss and clarify each of the recommendations. Further discussions were facilitated so attendees could consider the feasibility of implementing each recommendation without additional funding or staffing within 12–18 months.

Following the facilitated discussion, workshop attendees were asked to anonymously vote for their top three implementation priorities. The Zoom online polling function was used to vote for priorities 1, 2, 3 in knockout rounds. That is, each participant voted for their first priority from the list, and the recommendation with the highest vote was removed prior to voting for priority 2. This process was repeated for priority 3. After each polling round, results were automatically collated by the Zoom poll function and shared with the group. Participants were invited to discuss these results or any other points before the next voting round was opened. Participants could vote for the same item in subsequent rounds if their chosen priority was not selected.

## Results

### Survey 1: shortlisting priority recommendations

Seventy-five health professionals (81% female, median age 36) and 16 consumers (80% female, median age 51) responded to survey 1; responses are presented in Table 1. We could not calculate the response rate for health professionals because the invitations to participate (with the survey link attached) were emailed broadly via the Australian Stroke Coalition and state-based stroke clinical networks. The response rate for consumers was 43% (37 members of the Stroke Foundation Living Guidelines project consumer panel were invited to participate). In general, respondents perceived that implementing guideline recommendations was very important—the median score for importance of implementing each recommendation was 9 out of 10. Fourteen additional recommendations were nominated by respondents as being important to implement. Authors CL and KH reviewed these suggestions in terms of the strength of the evidence and whether change in practice could be measured. Of all these suggestions, only endovascular clot retrieval (suggested by 3 respondents) was deemed to have strong supporting evidence, having been included in the Stroke Guidelines for the first time in 2017, and has data routinely collected (via the Australian Stroke Clinical Registry). Endovascular clot retrieval was then added to the bundled recommendation regarding timely scan and access to thrombolysis *or endovascular clot retrieval* for eligible patients.

**Table 1:** Response to survey 1 regarding importance of guideline recommendations for implementation

Guideline recommendation	Stroke survivors, carers, family (*n* = 16)				Health professionals, researchers (*n* = 75)			
	Frequency of being top 5 most important (%)		Median importance	Mean importance	Frequency of being top 5 most important (%)		Median importance	Mean importance
Ambulances should take patients with suspected stroke to a hospital that has a stroke unit and can perform thrombolysis or clot retrieval	10	63%	10	9.8	37	49%	10	9.7
Patients with stroke who could possibly benefit from thrombolysis (clot-busting) should be assessed by the stroke team in the emergency department, given a brain scan (CT or MRI) within 60 minutes, and if found to be eligible, given thrombolysis within 4.5 hours	9	56%	10	9.3	33	44%	10	9.7
A carotid artery scan should be given to any patients whose stroke could have been caused by a clot in their carotid arteries	1	6%	9.5	8.5	6	8%	9	8.9
All patients with stroke should be admitted to hospital and be treated in a stroke unit with an interdisciplinary team, made up of medical, nursing and allied health professionals	9	56%	10	9.6	32	43%	10	9.4
Antiplatelet medication (aspirin, clopidogrel or dipyridamole) should be given as soon as the stroke is determined to be a clot and not a bleed, and if the patient is not receiving thrombolysis or clot retrieval	1	6%	9	8.7	7	9%	8	8.3
Blood glucose should be monitored for the first 72 hours, and medication given if the glucose levels are too high	0	0%	8.5	8.6	0	0%	8	8.0
Blood pressure-lowering medication should be given or increased for all patients with stroke and TIA who have blood pressure over 140/90 mmHg, before they are discharged from hospital	2	13%	9.5	8.8	8	11%	8	8.1
Antiplatelet medication (aspirin, clopidogrel or dipyridamole) should be prescribed to all people with ischaemic stroke or TIA who are not taking anticoagulants	2	13%	9	8.4	9	12%	8	8.1
Oral anticoagulation medication (blood thinners) should be prescribed for patients with ischaemic stroke and TIA who have atrial fibrillation (irregular heartbeat)	2	13%	9	8.8	10	13%	9	8.6
All people with an ischaemic stroke or TIA that may have been caused by an artery blocked by cholesterol plaque should be prescribed statins	0	0%	8	7.6	2	3%	8	7.8
Early supported discharge, which links hospital rehab with services for community and home rehab, should be offered to patients with mild to moderate stroke, if the appropriate services are available	0	0%	9.5	8.8	16	21%	10	8.9
Recovery goals should be set together with the stroke survivor, their family or carer, and the stroke team. The goals should be well-defined, specific and challenging, clearly documented, and reviewed and updated regularly	3	19%	9	8.8	13	17%	9	8.6
Out-of-bed activities should start within 48 hours of a patient's stroke, unless it is inappropriate (e.g. due to a patient being under palliative care)	3	19%	8	8.1	10	13%	9	8.5
Stroke survivors who have trouble walking should be given as many chances as possible to practice their walking repetitively and tailored to their needs	2	13%	9	8.4	8	11%	9	8.5
Constraint-induced movement therapy, in which someone's good hand is restrained so they have to use their affected hand, should be given to stroke survivors with some ability to move their wrists and fingers. It should involve a minimum of 2 hours of active therapy per day for 2 weeks, plus restraint of the good hand for at least 6 hours per day. A harness can also be used during therapy to restrain their torso	1	6%	6	6.6	2	3%	7	6.6
Stroke survivors who live at home and have trouble with their daily activities should be assessed by a trained clinician and given therapy, e.g. practising specific tasks and training to use aids and equipment	0	0%	8	8.1	10	13%	9	8.7
Speech and language therapy should be given to stroke survivors with aphasia, to improve their ability to communicate their wants and needs	3	19%	10	8.9	10	13%	9	8.9
All patients with stroke should be assessed and monitored for hydration problems, i.e. dehydration or over-hydration, and managed if necessary	0	0%	8	7.8	1	1%	8	8.3
Patients with stroke should be screened for malnutrition when first admitted and again at least every week while they are in hospital, with nutritional supplements given if they need them	0	0%	7.5	7.4	1	1%	7	7.3
Antidepressants should be considered for stroke survivors with symptoms of depression	0	0%	8	8	4	5%	8	8.1
All stroke survivors, their family and carers should be offered information that suits their individual needs and their language or communication requirements	5	31%	10	9.3	14	19%	10	9.2
A comprehensive discharge care plan that addresses the patient's specific needs should be developed together with them and their carer before they are discharged from hospital	3	19%	9.5	8.4	22	29%	10	9.1
Carers should be given tailored information and support at all stages of recovery, including opportunities to talk with the relevant health professionals about the stroke, what the stroke team does, test results, treatment and discharge plans, community services and contact details. It can be given before discharge or in the home, and can be face-to-face, over the phone or online	4	25%	9.5	8.5	15	2%	10	8.8

*CT* computed tomography, *MRI* magnetic resonance imaging, *TIA* transient ischaemic attack

There was clear consensus regarding the relative importance of the 23 recommendations, with nine recommendations ranked by both consumers and health professionals in the top 11 in terms of median and mean importance (ambulance to stroke hospital pathway; timely scanning and thrombolysis for eligible patients; treatment in a dedicated stroke unit; provision of discharge care plan; access to early supported discharge; carer support; information provision and aphasia therapy; goal-setting). No other recommendation scored within the top 10 for both consumers and health professionals, so the authorship team made the pragmatic decision to include the recommendation regarding prescription of oral anticoagulants (ranked 12th for both groups) in survey 2 because this was a relatively important recommendation to both groups, and allowed inclusion of a recommendation addressing secondary stroke prevention.

### Survey 2: ranking shortlisted recommendations

**Fig. 2:**
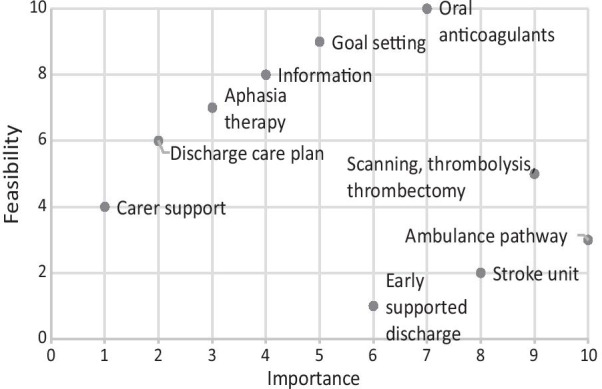
Scatter plot of aggregated rankings of shortlisted recommendations

Twenty-six health professionals and 12 consumers completed the second survey to rank the shortlisted priority recommendations in terms of importance and feasibility. The recommendations listed according to aggregate rankings of importance and feasibility are presented in Table 2 and are plotted in Fig. 2.

**Table 2:** Aggregated ranked lists from survey 2

	Importance ranking (10 = highest)	Feasibility ranking (10 = highest)
Ambulances should take patients with suspected stroke to a hospital that has a stroke unit and can perform thrombolysis or clot retrieval	10	3
Stroke patients should be assessed by the stroke team in the emergency department, given an urgent brain scan (within 30–60 minutes), and if found to be eligible, given thrombolysis (up to 9 hours from the stroke or midpoint of sleep) and/or thrombectomy (up to 24 hours after they were last known to be well)	9	5
All patients with stroke should be admitted to hospital and be treated in a stroke unit with an interdisciplinary team, made up of medical, nursing and allied health professionals	8	2
Oral anticoagulation medication (blood thinners) should be prescribed for patients with ischaemic stroke and TIA who have atrial fibrillation (irregular heartbeat)	7	10
Early supported discharge, which links hospital rehab with services for community and home rehab, should be offered to patients with mild to moderate stroke, if the appropriate services are available	6	1
Recovery goals should be set together with the stroke survivor, their family or carer, and the stroke team. The goals should be well-defined, specific and challenging, clearly documented, and reviewed and updated regularly	5	9
Speech and language therapy should be given to stroke survivors with aphasia, to improve their ability to communicate their wants and needs	4	7
All stroke survivors, their family and carers should be offered information that suits their individual needs and their language or communication requirements	3	8
A comprehensive discharge care plan that addresses the patient's specific needs should be developed together with them and their carer before they are discharged from hospital	2	6
Carers should be given tailored information and support at all stages of recovery, including opportunities to talk with the relevant health professionals about the stroke, what the stroke team does, test results, treatment and discharge plans, community services and contact details. It can be given before discharge or in the home, and can be face-to-face, over the phone or online	1	4

### Facilitated workshop: reaching consensus on priority recommendations

Ten participants (3 health professionals, 5 stroke survivors and 1 carer) attended the facilitated online meeting. After the presentations and facilitated discussion, four recommendations (early supported discharge, carer support, discharge care plan, aphasia therapy) that were illustrated on the scatter plot to have both comparatively less importance and lower feasibility than other recommendations were excluded from further consideration.

Discussions and anonymous online voting on the remaining six recommendations were facilitated by members of the research team who did not have voting rights; all decisions were made by the panel participants. One participant was unable to use the Zoom online polling function and so sent the vote for each round via the private chat function to author CL. The third round of voting resulted in a three-way tie, so the participants were asked to discuss the pros and cons of implementing each of the remaining recommendations, and ask the research team any questions. Following this discussion, a fourth round of polling was undertaken, and a clear third priority was identified. Table 3 shows results from each polling round and the top three prioritized recommendations.

**Table 3:** Results from anonymous polling

Recommendation	Round 1	Round 2	Round 3	Round 4
Ambulances should take patients with suspected stroke to a hospital that has a stroke unit and can perform thrombolysis or clot retrieval	3	1	3	
Stroke patients should be assessed by the stroke team in the emergency department, given an urgent brain scan (within 30–60 minutes), and if found to be eligible, given thrombolysis (up to 9 hours from the stroke or midpoint of sleep) and/or thrombectomy (up to 24 hours after they were last known to be well)	**4**			
All patients with stroke should be admitted to hospital and be treated in a stroke unit with an interdisciplinary team, made up of medical, nursing and allied health professionals	3	**8**		
Oral anticoagulation medication (blood thinners) should be prescribed for patients with ischaemic stroke and TIA who have atrial fibrillation (irregular heartbeat)		1	3	
Recovery goals should be set together with the stroke survivor, their family or carer, and the stroke team. The goals should be well-defined, specific and challenging, clearly documented, and reviewed and updated regularly				
All stroke survivors, their family and carers should be offered information that suits their individual needs and their language or communication requirements			3	**10**

Bolded figure is the recommendation with the highest polling for each round of voting

## Discussion

We have developed and described a rigorous process to identify high-priority guideline recommendations for implementation that involves consumers, health professionals and researchers, and we have illustrated its use in stroke management in Australia. Implementation of these recommendations will be addressed in a future piece of work. Our process is an important development for parties interested in large-scale guideline implementation projects, because identifying key priorities that are shared by different stakeholder groups can help to reveal a collective vision, overcome barriers and facilitate collective action towards successful implementation [14]. Although different methods have been used to identify priorities for implementation, such as modified Delphi in stroke and brain injury rehabilitation [15, 15], or applying implementation criteria to identify priorities in aphasia [17], we were unable to identify previously conducted guideline prioritization projects which have incorporated the perspectives of consumers. Methods used to set priorities for other purposes (e.g. priority-setting partnerships to identify research priorities [18], discrete choice experiments to prioritize health service innovation investments [19], conjoint analysis to prioritize innovations for implementation [3]) could not be applied to our project, given the size and scale of our Stroke Guidelines, and our desire to seek the priorities of the different stakeholder groups. We have demonstrated that use of this process is feasible, even with guidelines that have hundreds of recommendations, and even during a global pandemic which has seen the demise of face-to-face meetings.

While it is possible that some guideline recommendations important to clinicians and consumers may not have been included in the shortlisting process due to the expert-nominated prioritization criteria, there was opportunity for respondents from the wider stroke community to identify additional implementation priorities. This proved important in our case study, where the recommendation of endovascular clot retrieval was incorporated into the bundle of care regarding early scanning and thrombolysis following review of clinician and consumer responses. The effects of implementation strategies can only be assessed if performance of the intended practice can be measured, so for the purpose of this piece of work, only recommendations which could be measured, and further, were not being delivered as intended, were included for consideration. The criteria nominated by our expert panel are similar to criteria suggested by other researchers involved in prioritizing recommendations for implementation; practice recommendations underpinned by robust evidence are commonly considered the most important to address [4, 15–17]. This can hinder implementation efforts in clinical areas where a strong evidence base is lacking, therein highlighting the importance of recent work led by the James Lind Alliance to identify research priorities for different health conditions, which has incorporated the perspective of health professionals and consumers [18].

Importantly, our process does not predicate that any one criterion (such as “strong evidence”) should be used in every project. Our process is adaptable and explicitly requires tailoring for specific guidelines, stakeholder groups and contexts, through the first step of “identifying key implementation criteria” for each project. For this reason, we anticipate our process could be of use to others interested in identifying priorities for implementation for different health conditions in different world regions.

Our prioritization exercise will direct national implementation efforts to promote the delivery of evidence-based stroke care in Australia. Future work may determine whether the living approach to continually updating the Australian Stroke Clinical Guidelines impacts on these priorities and their implementation. We anticipate that having chosen recommendations with a strong evidence base, these recommendations will be unlikely to change as new studies are published.

A clear limitation of this work is that we have yet to evaluate whether it is possible to effectively implement these prioritized recommendations. Work is currently underway to develop, deliver and evaluate theory-informed strategies to implement the recommendations in stroke services across Australia. It is likely that this work will provide additional information to refine our process.

## Conclusion

This study demonstrates that it is possible to undertake a robust process that involves consumers, clinicians and researchers to systematically prioritize guideline recommendations for implementation. The process is generalizable and could be applied in clinical areas other than stroke and in different geographical regions to identify implementation priorities. The identification of three clear priority recommendations for implementation from the Australian Stroke Guidelines will directly inform the development and delivery of national implementation strategies.

## Supplementary Information

**Additional file 1: Appendix 1.** Methodology of Australian Clinical Guidelines for Stroke Management (Living Guidelines).

**Additional file 2: Appendix 2.** Survey 1: Rating relative importance of guideline recommendations.

## Data Availability

The datasets used and analysed during the current study are available from the corresponding author on reasonable request.

## Abbreviations

GRADE: Grading of recommendations, assessment, development and evaluation
TIA: Transient ischaemic attack
MRI: Magnetic resonance imaging
CT: Computed tomography
