# Supplementary material for: Prioritizing guideline recommendations for implementation: a systematic, consumer-inclusive process with a case study using the Australian Clinical Guidelines for Stroke Management
Source: Health Res Policy Syst. 2021 May 22;19:85. doi: 10.1186/s12961-021-00734-w (PMC8140744; doi:10.1186/s12961-021-00734-w)
Supplement: Supplementary file 1 — Additional file 1: Appendix 1. Methodology of Australian Clinical Guidelines for Stroke Management (Living Guidelines). [file 12961_2021_734_MOESM1_ESM.pdf]

Methodology of Australian Clinical Guidelines for Stroke Management (Living Guidelines)

Reproduced with permission from https://informme.org.au/en/Guidelines/Clinical-Guidelines-for-Stroke-Management

Development of questions

Questions have been extensively developed and reviewed over the four iterations of the guidelines. In this 'living' phase the Content Steering Group reviews the PICO (population, intervention/s, comparator, outcomes) questions on an annual basis.

Literature identification

On a monthly basis, Stroke Foundation staff monitor the literature for relevant, new evidence by screening all randomised controlled trials or systematic reviews related to stroke published in the Pubmed database. One member of the Stroke Foundation project team initially screens all abstracts and excludes clearly irrelevant studies. Potentially included studies are allocated to relevant topics covered by the guidelines and a second member of the project team reviews and confirms included studies prior to sending to the relevant working group members. In addition, each month new economic studies and studies related to patient values and preferences are also captured.

Clinical expert review

Where new evidence has been identified by the project team a summary is sent to content experts who review and make a final decision to include or exclude the study and also to assess the potential impact of the new evidence on current recommendations. As a result of this assessment one of two options will be communicated for each topic:

a. New evidence is unlikely to change current recommendations: review and potentially integrate information in the next review cycle; or

b. New relevant evidence may change current recommendations: rapidly review.

Data extraction, updating evidence summary and GRADE profile

For rapid updates, the project team incorporates the new evidence into the existing body of evidence by:

- Updating the Summary of Findings table including the risk of bias assessment
- Reviewing any additional studies related to Preferences and values of patients on the topic

Concurrently members of the economic working group review newly published economic studies.

The project team then drafts changes to the overall summary (GRADE profile). This profile is then reviewed and modified by clinical content experts and people with relevant lived experience (consumers). Finally changes to the changes to the recommendation, rationale and practical considerations are considered, discussed and agreed.

Draft changes are then circulated to the wider expert working groups (including consumer panel) for internal review. Once signed off by the Steering Group a period of public consultation is undertaken. Feedback is then reviewed and any changes made in response to feedback before finally submitting to the National Health and Medical Research Council (NHMRC) for approval.

Brief summary of GRADE

The Guidelines were developed following the GRADE methodology (Grading of Recommendations, Assessment, Development and Evaluation).

GRADE 'evidence to decision' framework includes a minimum of four factors to guide the development of a recommendation and determine the strength of that recommendation:

1. The balance between desirable and undesirable consequences.
2. Confidence in the estimates of effect (quality of evidence).
3. Confidence in values and preferences and their variability (clinical and consumer preferences).
4. Resource use (cost and implementation considerations).

For full details of how GRADE is used for developing clinical recommendations, refer to the GRADE handbook, available at: http://gdt.guidelinedevelopment.org/app/handbook/handbook.html.

Strength of recommendations

The GRADE process uses only two categories for the strength of recommendations, based on how confident the guideline panel is that the "desirable effects of an intervention outweigh undesirable effects […] across the range of patients for whom the recommendation is intended" (GRADE Handbook):

- **Strong** recommendations: where guideline authors are certain that the evidence supports a clear balance towards either desirable or undesirable effects; or
- **Weak** recommendations: where the guideline panel is uncertain about the balance between desirable and undesirable effects

These strong or weak recommendations can either be for or against an intervention. If the recommendation is against an intervention this means it is recommended NOT to do that intervention. There are a number of recommendations where we have stated that the intervention may only be used in the context of research. We have done this because these are guidelines for clinical practice, and while the intervention cannot be recommended as standard practice at the current time, we recognise there is good rationale to continue further research.

The implications of a strong or weak recommendation for a particular treatment are summarised in the GRADE handbook as follows:

Table 1: Implications of GRADE recommendation categories (for a positive recommendation) for patients, clinicians and policy makers. Source: GRADE Handbook (http://gdt.guidelinedevelopment.org/app/handbook/handbook.html)

	**Strong recommendation**	**Weak recommendation**
**For patients**	Most individuals in this situation would want the recommended course of	The majority of individuals in this situation would want the suggested course of action, but many would not

	action and only a small proportion would not.	
**For clinicians**	Most individuals should receive the recommended course of action. Adherence to this recommendation according to the guideline could be used as a quality criterion or performance indicator. Formal decision aids are not likely to be needed to help individuals make decisions consistent with their values and preferences.	Recognise that different choices will be appropriate for different patients, and that you must help each patient arrive at a management decision consistent with her or his values and preferences. Decision aids may well be useful helping individuals making decisions consistent with their values and preferences. Clinicians should expect to spend more time with patients when working towards a decision.
**For policy makers**	The recommendation can be adapted as policy in most situations including for the use as performance indicators.	Policy making will require substantial debates and involvement of many stakeholders. Policies are also more likely to vary between regions. Performance indicators would have to focus on the fact that adequate deliberation about the management options has taken place.

For topics where there is either a lack of evidence or insufficient quality of evidence on which to base a recommendation but the guideline panel believed advice should be made, statements were developed based on consensus and expert opinion (guided by any underlying or indirect evidence). These statements are labelled as ‘Practice statements’ and correspond to 'consensus-based recommendations' outlined in the NHMRC procedures and requirements.

For topics outside the search strategy (i.e. where no systematic literature search was conducted), additional considerations are provided. These are labelled ‘Info Box’ and correspond to ‘practice points’ outlined in the NHMRC procedures and requirements.

Explanation of absolute effect estimates used

The standardised evidence profile tables presented in the Clinical Guidelines include “Absolute effect estimates” for dichotomous outcomes. These represent the number of people per 1000 people expected to have the outcome in the control and intervention groups. This estimated risk in people receiving the intervention is based on a relative effect estimate which might be adjusted, e.g. to account for baseline differences between participants or when effect estimates have been pooled from different studies in a systematic review and adjusted to account for the variance of each individual estimate. Therefore, this estimated risk in the intervention group may differ from the raw

estimate of the intervention group risk from the corresponding study. The estimated risk reflects the best estimate of the risk in the relevant population, relative to the risk observed among patients receiving the control or comparator intervention.

Wherever possible (i.e. when the relevant study reported enough information to allow the calculation to be done), these estimates were calculated using the following procedure:

1. Obtain the relative effect estimate (odds ratio or relative risk) and confidence interval from the best available study (systematic review or primary study) providing evidence about the effects of the intervention.
2. Use the observed number of events in the control group of the same study to calculate a baseline risk per 1000 people (or "assumed control risk").
3. Calculate an estimate of the corresponding risk per 1000 in people receiving the intervention using the relative effect estimate. This can be done using methods based on the formulas for calculating absolute risk reductions provided in the *Cochrane Handbook for Systematic Reviews of Interventions* (http://handbook.cochrane.org/). Applying the same calculations to the upper and lower bounds of the confidence interval for the relative effect estimate gives a confidence interval for the risk in the intervention group, which is then used to calculate the confidence interval for the difference per 1000 people, reported in the evidence tables.

Cost effectiveness summaries

There are several important points to consider when interpreting the cost-effectiveness information provided in the *Resources and Other Considerations* sections of the Clinical Guidelines.

Firstly, an intervention can be cost-effective without being cost-saving. This means that although there is an additional cost for the health benefits gained from the intervention, the intervention is still considered worthwhile. The incremental cost-effectiveness ratios (ICER) presented (e.g. cost per quality adjusted life year gained) are an indication of the cost-effectiveness or "value-for-money", with lower ICERs indicating better cost-effectiveness of an intervention.

Secondly, whether or not the intervention is cost-effective is a judgment call; and should reflect a society's willingness-to-pay to have the intervention for the potential outcomes achieved. An ICER that is approximately or equivalent to US$50,000 has been commonly used by researchers in the past as a threshold for judging an intervention as being cost-effective (http://www.nejm.org/doi/full/10.1056/NEJMp1405158#t=article). However, no scientific basis for this threshold exists and actual willingness-to-pay may differ. For example, in a survey of 1000 Australian respondents conducted in 2007, the willingness-to-pay for an additional quality adjusted life year in Australia was estimated to be $64,000 (https://www.ncbi.nlm.nih.gov/pubmed/19382128).

Thirdly, there is no absolute threshold for determining whether an intervention should be funded based on the ICER (https://www.ncbi.nlm.nih.gov/pmc/articles/PMC5153921/). ICERs are only one

of the major factors considered in priority setting (the process to decide which interventions should be funded within a given resource constraint). Other considerations include affordability, budget impact, fairness, feasibility and other factors that are important in the local context (https://www.ncbi.nlm.nih.gov/pmc/articles/PMC5153921/).

Lastly, in areas where there are no data from economic evaluations that support the recommendations or practice statements, it remains unclear whether the additional costs of providing the intervention above usual care for the additional potential benefits obtained is justified. However, this should not detract from implementing the Clinical Guideline recommendations.

Use of language related to timing of interventions

***Immediate*:** without delay, or within minutes, not hours (life critical action required).
***Urgent*:** minutes to several hours (immediate action but not life critical).
Very early: within hours and up to 24 hours.
Early: within 48 hours.

For all Clinical Guideline recommendations we make the assumption that healthcare professionals will be appropriately qualified and skilled to carry out the intervention.
