# Supplementary material for: Prioritizing guideline recommendations for implementation: a systematic, consumer-inclusive process with a case study using the Australian Clinical Guidelines for Stroke Management
Source: Health Res Policy Syst. 2021 May 22;19:85. doi: 10.1186/s12961-021-00734-w (PMC8140744; doi:10.1186/s12961-021-00734-w)
Supplement: Supplementary file 2 — Additional file 2: Appendix 2. Survey 1: Rating relative importance of guideline recommendations. [file 12961_2021_734_MOESM2_ESM.docx]

*survey: TO BE ADMINISTERED VIA SURVEY MONKEY*

**Stroke foundation priorities survey**

There are a lot of recommendations in the Clinical Guidelines for Stroke Management. However, there are evidence-practice gaps (i.e., gaps between the findings from research evidence and actual clinical practice) in almost all aspects of stroke care. This means that the recommendations are not always being implemented properly, and that some people miss out on recommended care.

The Stroke Foundation is committed to put extra efforts in to support how care is delivered in line with guideline recommendations – this project is about choosing which ***guideline recommendations are most important to people with stroke, their families and health professionals/researchers***.

There is currently no information about what people with stroke and their families think are the most important evidence-practice gaps to address.

This survey aims to identify clinicians’ opinions about which recommendations from the Stroke Foundation’s Clinical Guidelines for Stroke Management are ***most important*** to implement.

Instructions

The Stroke Foundation has worked out which guideline recommendations are based on the strongest evidence. Other than being backed by good research, ***what do you think makes a particular guideline recommendation important to be implemented?***

We are seeking your opinions on which of these recommendations are most important to implement.

Please think about each recommendation carefully.

*Importance* = This is your personal opinion about whether the recommendation is important to implement in practice.

In order to understand why you have rated recommendations as very important (7 or above) or not important (3 or below), you will be prompted to provide a reason for your score. This will help us understand what factors influenced your decisions.

Some of these guideline recommendations have been simplified for the purposes of this survey. We have included information about the proportion of people affected by each recommendation, and the potential outcome, to assist you with your decision-making.

| No. | Topic | Recommendations | How many people might this impact? | What is the size of the impact? | Importance  (rate 1 not important at all to 10 extremely important) | Reason  (prompt if response 7 or above/3 or below) |
| --- | --- | --- | --- | --- | --- | --- |
| 1. | Prehospital care | All stroke patients should be admitted to hospital and be treated in a stroke unit with an interdisciplinary team.  Ambulances should take suspected stroke patients to a hospital that has a stroke unit and can perform thrombolysis or clot retrieval | *75% of people arrive by ambulance* | *can reduce death and disability* |  |  |
| 2. | Thrombolysis | Stroke patients who could possibly benefit from thrombolysis (clot-busting) should be assessed by the stroke team in the emergency department, given a brain scan (CT or MRI) within 60 minutes, and if found to be eligible, given thrombolysis within 4.5 hours of their stroke | *15-20% of people eligible for thrombolysis* | *can reduce death and disability by 5-11%* |  |  |
| 3. | Vascular imaging | A carotid artery scan should be given to any patients whose stroke could have been caused by a clot in their carotid arteries | *25-30% of strokes caused by carotid disease* | *management of carotid disease can reduce risk of future stroke* |  |  |
| 4. | Stroke unit care | All stroke patients should be admitted to hospital and be treated in a stroke unit with an interdisciplinary team, made up of medical, nursing and allied health professionals. | *100% of patients with strokes eligible* | *can reduce death and disability by 6%* |  |  |
| 5. | Acute antithrombotic therapy | Antiplatelet medication (aspirin, clopidogrel or dipyridamole) should be given as soon as the stroke is determined to be a clot not a bleed, and if they're not getting thrombolysis or clot retrieval. | *70% of strokes eligible - ischaemic without thrombolysis* | *can reduce death, disability & future strokes by 1%* |  |  |
| 6. | Acute glycaemic therapy | Blood glucose should be monitored for the first 72 hours, and medication given if the glucose levels are too high. | *20-30% have glucose issues in acute stroke* | *can reduce death and disability by 16% when combined with management of fever and swallowing* |  |  |
| 7. | Long-term blood pressure management | Blood pressure lowering medication should be given or increased for all stroke and TIA patients with blood pressure over 140/90 mmHg, before they are discharged from hospital. | *75% of strokes have high blood pressure* | *can reduce future strokes by 1.45%* |  |  |
| 8. | Long-term antiplatelet therapy | Antiplatelet medication (aspirin, clopidogrel or dipyridamole) should be prescribed to all people with ischaemic stroke or TIA who are not taking anticoagulants. | *80% strokes are ischaemic* | *can reduce future strokes by 1.8%* |  |  |
| 9. | Long-term anticoagulant therapy | Oral anticoagulation medication (blood thinners) should be prescribed for ischaemic stroke and TIA patients who have atrial fibrillation (irregular heartbeat). | *20% of strokes are related to atrial fibrillation* | *can reduce future strokes by 5.24%* |  |  |
| 10. | Cholesterol lowering therapy | All people with an ischaemic stroke or TIA that may have been caused by an artery blocked by cholesterol plaque should be prescribed statins. | *80% strokes are ischaemic* | *can reduce future strokes by 1.3%* |  |  |
| 11. | Early supported discharge | Early supported discharge, which links hospital rehab with services for community and home rehab, should be offered to patients with mild to moderate strokes, if the appropriate services are available. | *20-30% eligible - with mild to moderate strokes* | *can reduce death and disability by 8%* |  |  |
| 12. | Goal setting | Recovery goals should be set together with the stroke survivor, their family or carer, and the stroke team. The goals should be well-defined, specific and challenging, clearly documented, and reviewed and updated regularly. | *100% of strokes* | *benefit unclear but may benefit quality of life, activities of daily living, function, length of stay and self-efficacy* |  |  |
| 13. | Early mobilisation | Out-of-bed activities should start within 48 hours of a patient's stroke, unless it's inappropriate (e.g. due to a patient being under palliative care). | *90% able to mobilise post stroke* | *can lead to small reduction in long-term disability* |  |  |
| 14. | Walking | Stroke survivors who have trouble walking should be given as many chances as possible to practice their walking repetitively and tailored to their needs. | *56% unable to walk on admission* | *can lead to small improvement in walking ability* |  |  |
| 15. | Upper limb activity | Constraint-induced movement therapy, in which someone's good hand is restrained so they have to use their affected hand, should be given to stroke survivors with some ability to move their wrists and fingers. It should involve a minimum of 2 hours active therapy per day for 2 weeks, plus restraint of the good hand for at least 6 hours per day. A harness can also be used during therapy to restrain their torso. | *10% of strokes eligible* | *can lead to small improvement in arm function* |  |  |
| 16. | Activities of daily living | Stroke survivors who live at home and have trouble with their daily activities should be assessed by a trained clinician and given therapy, e.g. practising specific tasks and training to use aids and equipment. | *87% of strokes have issues with activities of daily living* | *can reduce death and disability by 2.6%* |  |  |
| 17. | Communication | Speech and language therapy should be given to stroke survivors with aphasia, to improve their ability to communicate their wants and needs. | *25-33% have aphasia* | *can lead to small to moderate improvement in functional communication* |  |  |
| 18. | Early hydration | All stroke patients should be assessed and monitored for hydration problems, i.e. dehydration or over-hydration, and managed if necessary. | *15% have hydration problems* | *can reduce death, disability, and pneumonia* |  |  |
| 19. | Early feeding | Stroke patients should be screened for malnutrition when first admitted and again at least every week while they're in hospital, with nutritional supplements given if they need them. | *10-20% have malnutrition* | *benefit uncertain but may lead to small reduction in deaths* |  |  |
| 20. | Mood disturbance | Antidepressants should be considered for stroke survivors with symptoms of depression. | *30% develop depression* | *may lead to moderate reduction of depression* |  |  |
| 21. | Information and education | All stroke survivors, their family and carers should be offered information that suits their individual needs and their language or communication requirements. | *100% of strokes* | *Improves patient and carer knowledge* |  |  |
| 22. | Discharge care plans | A comprehensive discharge care plan that addresses the patient's specific needs should be developed together with them and their carer before they're discharged from hospital. | *100% of strokes* | *can lead to 3% reduction in readmissions* |  |  |
| 23. | Carer support | Carers should be given tailored information and support at all stages of recovery, including opportunities to talk with the relevant health professionals about the stroke, what the stroke team does, test results, treatment and discharge plans, community services, and contact details. It can be given before discharge or in the home, and can be face-to-face, over the phone or online. | *30-40% have carers* | *can lead to small reduction in carer stress and strain* |  |  |

Which best describes you: tick all that apply

I am a stroke survivor

I am a carer of a stroke survivor

I am a family member of a stroke survivor

I am a health professional working with stroke survivors

I am a researcher working with stroke survivors

About you: please list

• Age:

• Sex:

• Profession:

• Location (City/State):

Are there any other recommendations, not included on this list, that you believe are an important priority to improve in practice? If yes, please state which recommendation and your reasoning for including it.

__________________________________________________________________________________

In order for us to contact you for the next stage of the research, can you please provide your contact details – we will not share these details with anyone else

__________________________________________________________________________________

Please indicate whether you would be willing to attend a face-to-face meeting in Melbourne in 2020 to participate in the final stage of this project (travel costs will be reimbursed)

I am interested in attending a face-to-face workshop

I am not interested in attending a face-to-face workshop

Thank you for completing this survey.
